# Psychometric Properties of the Chinese Version of the Child-to-Parent Violence Questionnaire in Adolescents with Attention-Deficit/Hyperactivity Disorder

**DOI:** 10.3390/children12040408

**Published:** 2025-03-24

**Authors:** Ching-Shu Tsai, Chung-Ying Lin, Ray C. Hsiao, Cheng-Fang Yen

**Affiliations:** 1 Department of Child and Adolescent Psychiatry, Chang Gung Memorial Hospital, Kaohsiung Medical Center, Kaohsiung 83341, Taiwan; jingshu@cgmh.org.tw; 2 College of Medicine, Chang Gung University, Taoyuan City 33302, Taiwan; 3 Institute of Allied Health Sciences, College of Medicine, National Cheng Kung University, Tainan 70101, Taiwan; cylin36933@gs.ncku.edu.tw; 4 Biostatistics Consulting Center, National Cheng Kung University Hospital, College of Medicine, National Cheng Kung University, Tainan 70101, Taiwan; 5 Department of Public Health, College of Medicine, National Cheng Kung University, Tainan 70101, Taiwan; 6 Department of Occupational Therapy, College of Medicine, National Cheng Kung University, Tainan 70101, Taiwan; 7 Department of Psychiatry and Behavioral Sciences, University of Washington School of Medicine, Seattle, WA 98195, USA; rhsiao@u.washington.edu; 8 Department of Psychiatry, Seattle Children’s, Seattle, WA 98105, USA; 9 Department of Psychiatry, Kaohsiung Medical University Hospital, Kaohsiung 80708, Taiwan; 10 Department of Psychiatry, School of Medicine, College of Medicine, Kaohsiung Medical University, Kaohsiung 80708, Taiwan

**Keywords:** adolescents, attention-deficit/hyperactivity disorder, child-to-parent violence, psychometric, questionnaire, validation

## Abstract

Purpose: Child-to-parent violence (CPV) is a major concern for adolescents with attention-deficit/hyperactivity disorder (ADHD). The Child-to-Parent Violence Questionnaire (CPV-Q) is a valid instrument for assessing a wide variety of CPV behaviors and the reasons; however, the psychometric properties of the Chinese version of CPV-Q (C-CPV-Q) in adolescents with ADHD have not been examined yet. This study examined the psychometric properties of both child and parent versions of C-CPV-Q in adolescents with ADHD. Participants and Methods: In total, 247 adolescents with ADHD and their parents participated in the study. Confirmatory factor analyses (CFAs) were conducted to examine the factor structure for CPV types and reasons. Internal consistency, cross-validation, and concurrent validity of the parent and child versions of C-CPV-Q were also evaluated. Results: The results of CFA found that both child and parent versions of the C-CPV-Q contained four domains of CPV types and two domains of CPV reasons in adolescents with ADHD. The correlations between parent and child reports of the C-CPV-Q assessing the same factors were stronger than those assessing different factors. Concurrent validity of the C-CPV-Q was supported by their positive correlations with internalizing, externalizing, attention deficit/hyperactivity, oppositional defiant, and conduct problems. Except for the financial aggression factor, the entire C-CPV-Q had acceptable internal consistency. Conclusions: The results indicate that the child and parent versions of the C-CPV-Q are valid instruments for assessing a wide variety of CPV behaviors and the reasons among adolescents with ADHD.

## 1. Introduction

Child-to-parent violence (CPV) is an important health and behavioral issue among adolescents in community and clinical units [1]. A review study on 145 publications demonstrated that the field of CPV is rapidly growing and doubling in the last decade [2]. Approximately 5% to 21% of children exhibited a certain form of physical aggression toward their parents in the general population [3]. A systematic review and meta-analysis indicated that 23% to 25% of children and adolescents in Latin America returned to psychological violence and 5% to 6% resorted to physical violence against their parents [4]. A study conducted in Australia also reported that 7% of the respondents (aged 14 to 25 years) had physically attacked their parents [5]. These findings suggest that CPV in adolescents is not uncommon. CPV can be a precursor to various forms of violent crime [1]. An analysis of crime cases in Spain revealed that adolescents who attack their parents are often the most serious offenders among minors [6]. CPV directly causes a loss of parental discipline and feelings of humiliation, which may compromise the safety of family members [7,8,9]. Therefore, CPV in adolescents needs to be investigated and prevented. These findings underscore the necessity of further investigation of CPV in adolescents. Multiple child (e.g., emotional insecurity, antisocial personality, and justification of violence), family (e.g., exposure of children to violence within the family, problematic parenting, and parental alcohol use), and school factors (e.g., children’s involvement in schools and friendships and teachers’ help in schools) significantly correlate with the risk of CPV in adolescents [10,11,12,13]. Alternatively, CPV has been considered a behavioral indicator of childhood distress or developmental needs [14]. For example, a study found that adolescents who exerted CPV presented higher rates of adverse childhood experiences than those without CPV; aggressors with cumulative adverse childhood experiences presented more insecure parental attachment, lower resilience, and lower emotional intelligence than those without cumulative ACEs [15]. A review study identified the need for further research into CPV including what makes interventions effective, what needs and outcomes the interventions are addressing, and the implications of classifications of CPV [16].

Several instruments have been developed for assessing the experiences of CPV, such as the Conflict Tactic Scale [17], Adolescents’ Parent-Directed Aggression Measure [18], Intra-family Violence Scale [19], Child-to-parent Aggression Questionnaire [20], and Child-to-Parent Violence Questionnaire (CPV-Q) [21,22]. Compared with other instruments for evaluating CPV, the CPV-Q has the following characteristics [21,22]. First, the CPV-Q evaluates multifaceted CPV, including psychological aggression, physical aggression, financial demands, and control/domination. In particular, other scales did not include control and domain over parents for assessment. Second, in addition to evaluating the types and frequencies of CPV, the CPV-Q assessed the reasons for CPV. Third, the CPV-Q has both child and parent versions for evaluating CPV from various informants. Studies have supported the reliability and validity of the child and parent versions of the CPV-Q [22]. A study comparing 11 instruments for evaluating CPV based on consensus-based standards for the selection of health measurement instruments concluded that the CPV-Q was of high quality [1].

The psychometric properties of the CPV-Q have not been examined among adolescents with a diagnosis of attention-deficit/hyperactivity disorder (ADHD). Involvement in violence is a major concern for adolescents with ADHD. A systematic review reported that adolescents with ADHD are at a higher risk of domestic and intimate partner violence during adulthood compared with those without ADHD [23]; however, few studies have examined CPV in children and adolescents with ADHD [2]. A study on Iranian parents of adolescents with ADHD reported that more than half of these parents had experienced at least one physical, verbal, psychological, or materialistic attack by their children [24]. Given the importance of evaluating CPV in adolescents with ADHD, this study examined the psychometric properties of both child and parent versions of Chinese version of the CPV-Q (C-CPV-Q) for assessing CPV in adolescents with ADHD. Specifically, this study examined the factor structure, internal consistency, cross-validation, and concurrent validity of the child and parent versions of C-CPV-Q.

## 2. Methods

### 2.1. Participants

In this cross-sectional study, we distributed surveys to adolescents with ADHD and their primary caregivers. Adolescents with ADHD from six child psychiatry outpatient clinics at two hospitals in Taiwan were included for analysis. Adolescents with ADHD meeting the following criteria were included in the study: (1) being 11–18 years of age and (2) having received a diagnosis of ADHD by a certified child psychiatrist in accordance with the *Diagnostic and Statistical Manual of Mental Disorders*, *Fifth Edition*, *Text Revision* [25]. Adolescents who had comorbid intellectual disability, severe autism spectrum disorder, bipolar disorder, schizophrenia, or any other cognitive deficits that may impede their understanding of the study’s purpose and completion of the research questionnaire were excluded, along with their parents.

### 2.2. Procedure

Three child psychiatrists reviewed the medical records of adolescents with ADHD who visited the selected outpatient clinics between August 2023 and July 2024. A total of 259 adolescents with ADHD and their parents were consecutively approached. In total, 247 adolescents with ADHD and their parents agreed to participate in the study. All participants were assured that their responses would remain confidential and that their participation or nonparticipation would not influence their right to receive medical services. This study was approved by the institutional review boards of two university-affiliated hospitals. Informed consent was obtained from all adolescents and their parents.

### 2.3. Measures

#### 2.3.1. Chinese Version of the CVP-Q

We used the Chinese version of the CVP-Q (C-CPV-Q) to evaluate CPV among adolescents in the year preceding the evaluation [21,22]. The C-CPV-Q is divided into two parts. The first part (C-CPV-Q-I) consists of 14 items that evaluate four domains of CPV, namely psychological aggression (four items, e.g., “I have told my parents ‘I hate you!’ and ‘I wish you were dead’’’), physical aggression (three items, e.g., “I have thrown things at my parents”), financial demand (three items, e.g., “I have demanded that my parents buy me things I know they cannot afford”), and control and domination (four items, e.g., “I have told my parents that at home they have to do what I want”). Each item is rated on a 5-point scale with endpoints of 0 (*never*), 1 (*once*), 2 (*two to three times*), 3 (*four to five times*), and 4 (*six times or more*). The second part (C-CPV-Q-II) consists of eight items evaluating two main categories of reasons for CPV, namely instrumental reasons (e.g., “To be able to come home later when going out at night”) and reactive reasons (e.g., “In response to a parents’ physical aggression”). In this study, we modified the item “to get more money from your father/mother” into two items evaluating the purposes of getting money for the purchase of 3C products such as cell phones or game points, or not. We also added another four reasons for CPV according to our clinical experience, namely in response to parents’ restricting their children’s use of 3C products, in response to parents’ different treatment styles with their children, in response to parents restricting their children’s social communication or intimate relationships, and in response to parents’ control of how their children dress. Finally, there were 13 items on the C-CPV-Q-II. Each reason was rated on a 4-point scale, with endpoints ranging from 0 (*never*) to 3 (*always*).

Adolescents and their parents were invited to complete the C-CPV-Q. Both child and parent versions of the questionnaire had the same content but differed only in their phrasing style. For example, the third item was phrased as “I have made negative, offensive, and/or degrading comments to my parents” in the child version but as “My child has made negative, offensive, and/or degrading comments to me” in the parent version.

#### 2.3.2. Child Behavior Checklist for Ages 6–18

We used the 112-item, parent-reported Chinese version of the Child Behavior Checklist for Ages 6–18 to evaluate adolescents’ behavioral problems [26,27,28]. We also used the recommended T-score transformations of raw behavior scores, which were adjusted for age and sex differences in behavior found in normative samples. In addition, we used the following domains for analysis: ADHD symptoms, internalizing problems (evaluated using scales for anxiety/depression, withdrawal/depression, and somatic syndrome disorder), externalizing problems (evaluated using scales for oppositional defiant [ODD] and conduct symptoms), attention deficit/hyperactivity problems, ODD problems, and conduct problems. This checklist has an internal consistency (Cronbach’s α) of 0.55–0.90 and a 1-month test–retest reliability (Pearson’s *r*) of 0.51–0.74, along with high construct validity (eight-factor structure) [29,30].

### 2.4. Data Analysis

Four sets of confirmatory factor analyses (CFAs) were conducted to examine the factor structure of the four measures: C-CPV-Q-I parent report, C-CPV-Q-I child report, C-CPV-Q-II parent report, and C-CPV-Q-II child report. For parent and child reports of C-CPV-Q-I, a four-factor structure was examined: items 1 to 4 as the psychological aggression factor; items 8, 10, and 11 as the physical aggression factor; items 6, 7, and 12 as the financial aggression factor; items 5, 9, 13, and 14 as the control/domain factor. For parent and child reports of C-CPV-Q-II, a two-factor structure was examined: items 1 to 5 as the instrumental reason factor; and items 6 to 12 as the reactive reason factor. For all the CFAs, Weight Least Square with Mean and Variance (WLSMV) estimator was used, and the following fit indices were used to decide if the factor structure is supported: comparative fit index (CFI) > 0.9, Tucker–Lewis index (TLI) > 0.9, root mean square error of approximation (RMSEA) < 0.08, and standardized root mean square residual (SRMR) < 0.08 [31]. Apart from the fit indices examining the entire factor structure, each item was evaluated using its factor loading, and a factor loading > 0.3 indicated that an item could be retained in its embedded factor [32].

After ensuring the factor structure, multigroup CFA was used to examine measurement invariance of the four measures across age subsamples (medium age or below vs. above medium age). In the multigroup CFA, three nested models were compared: (i) a configural model that freely estimated factor loadings and item intercepts for different subsamples; (ii) a metric invariance model that constrained factor loadings to be equal across subsamples; and (iii) a scalar invariance model that constrained both factor loadings and item intercepts to be equal across subsamples [33]. Measurement invariance was supported when the CFI difference and RMSEA difference between every two nested models was <0.01 [34].

Internal consistency of each factor for both measures and the entire set of measures was calculated using McDonald’s ω and Cronbach’s α, and a value (either ω or α) > 0.7 indicated acceptable levels of internal consistency [35]. Pearson correlations with a heatmap were then illustrated to demonstrate the cross-validation between C-CPV-Q-I and C-CPV-Q-II. Specifically, the same factors measured using different methods (i.e., parent or child reports) should have a higher correlation coefficient than different factors measured using different methods [36]. Apart from the cross-validation, concurrent validity of the C-CPV-Q-I and C-CPV-Q-II was evaluated using the following external criterion measures: internalizing problems, externalizing problems, attention deficit/hyperactivity problems, oppositional defiant problems, and conduct problems. According to Cohen’s recommendation, a correlation of 0.1 indicates a small magnitude; 0.3 indicates a moderate magnitude; and 0.5 indicates a large magnitude [37].

The CFAs and internal consistency were analyzed using R software via the *lavaan* 0.6-11 or *psych* 2.2.5 [38,39] packages. The heatmap with Pearson correlations was generated using *jamovi* (version 2.5) software [40].

## 3. Results

Among the 247 parents (mean [*SD*] age = 46.40 [6.41] years), slightly over one-fourth were male (*n* = 65; 26.3%), and nearly one-third had an educational level of high school or below (*n* = 80; 32.4%). With regard to the 247 children (mean [*SD*] age = 13.21 [2.03] years), most of them were boys (*n* = 206; 83.4%) (Table 1).

Table 2 demonstrates the factor loadings and fit indices for the C-CPV-Q-I in a four-factor structure (including psychological aggression, physical aggression, financial demand, and control/domination). Both parent and child reports had satisfactory fit indices: CFI = 0.938, TLI = 0.918, RMSEA (90% CI) = 0.025 (0.000, 0.047), and SRMR = 0.075 (parent report); CFI = 1.000, TLI = 1.191, RMSEA (90% CI) = 0.000 (0.000, 0.028), and SRMR = 0.080 (child report). All factor loadings were greater than 0.3 for both parent and child reports. In addition, the entire C-CPV-Q-I had acceptable internal consistency (ω/α = 0.89/0.85 for the parent report; =0.81/0.76 for the child report). Some items had weak factor loadings (e.g., Item 11 [factor loading = 0.302], Item 6 [factor loading = 0.304], and Item 12 [factor loading = 0.313]). Although these factor loadings were greater than the cut-off of being retained in their embedded factor (>0.3), they were weaker than other factor loadings found in this study. Moreover, the financial aggression factor in both child and parent reports had unsatisfactory internal consistency: ω/α = 0.45/0.41 for the parent report; =0.46/0.37 for the child report.

Table 3 presents the factor loadings and fit indices for the C-CPV-Q-II in a two-factor structure (including instrumental and reactive reasons). Both parent and child reports had satisfactory fit indices: CFI = 0.928, TLI = 0.905, RMSEA (90% CI) = 0.042 (0.019, 0.061), and SRMR = 0.078 (parent report); CFI = 0.938, TLI = 0.918, RMSEA (90% CI) = 0.025 (0.000, 0.048), and SRMR = 0.070 (child report). All factor loadings were greater than 0.3 for both parent and child reports. In addition, the entire C-CPV-Q-II had acceptable internal consistency (ω/α = 0.89/0.86 for the parent report; =0.87/0.84 for the child report).

Table 4 shows that the factor structure of the CPV-Q-I was invariant across different age subsamples in the present study for both children and parents; however, CPV-Q-II was not invariant across different age subsamples for both children and parents. Specifically, the metric invariance of the CPV-Q-II was not met, indicating that participants at different ages may interpret the CPV-Q-II differently.

**Figure 1:**
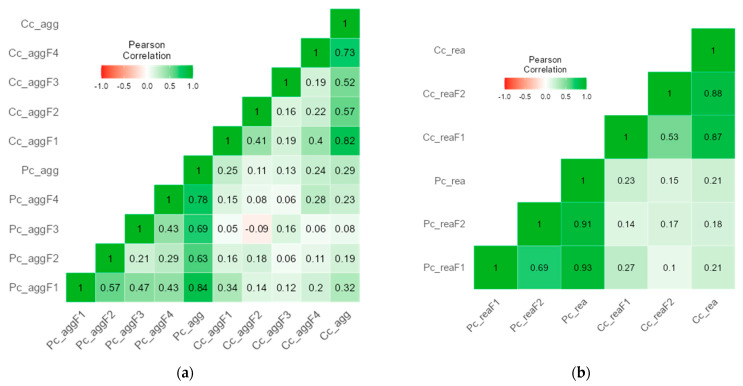
Cross-validation for the first and second parts of the Child-to-Parent Violence Questionnaire (CPV-Q-I of CPV-Q-II) between parent and child reports. Note: Pc_agg = the first part of Child-to-Parent Violence Questionnaire parent report; Cc_agg = the first part of the Child-to Parent Violence Questionnaire child report; aggF1 = Psychological Aggression factor; aggF2 = Physical Aggression factor; aggF3 = Financial Aggression factor; aggF4 = Control/Domain factor; Pc_rea = the second part of the Child-to-Parent Violence Questionnaire parent report; Cc_rea = the second part of the Child-to-Parent Violence Questionnaire child report; reaF1 = Instrumental Reason factor; reaF2 = Reactive Reason factor. (**a**) with CPV-Q-I, (**b**) with CPV-Q-II.

In terms of cross-validation (Figure 1), both C-CPV-Q-I and C-CPV-Q-II showed good properties. Specifically, the correlations between parent and child reports of C-CPV-Q-I assessing the same factors (e.g., 0.34 of parent-reported psychological aggression with child-reported psychological aggression) were stronger than those between parent and child reports assessing different factors (e.g., 0.05 to 0.16 for the correlations of parent-reported psychological aggression with child-reported physical aggression, financial aggression, and control/domain; Figure 1a). Similarly, the correlations between parent and child reports of C-CPV-Q-II assessing the same factors (e.g., 0.27 of parent-reported instrumental reasons with child-reported instrumental reasons) were stronger than those between parent and child reports assessing different factors (e.g., 0.14 for the correlation of parent-reported instrumental reasons with child-reported reactive reasons; Figure 1b).

**Figure 2:**
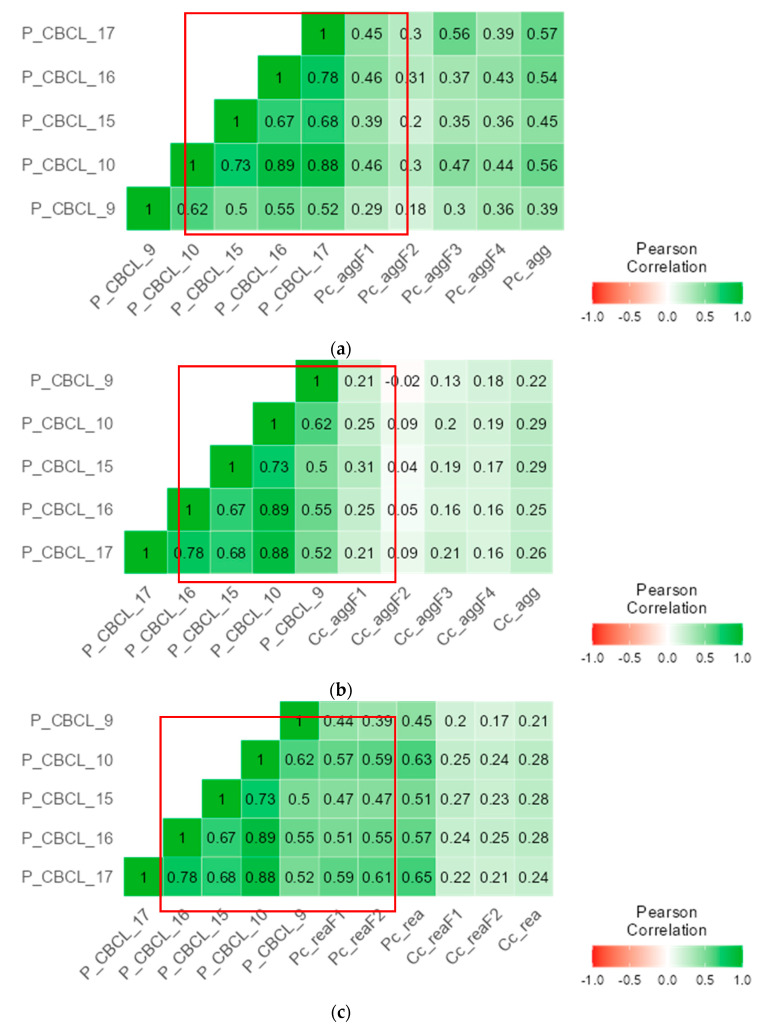
Concurrent validity of the first part of the Child-to-Parent Violence Questionnaire (CPV-Q-I) and the second part of the Child-to-Parent Violence Questionnaire (CPV-Q-II). Note. Red box indicates how CPV-Q-I and CPV-Q-II associate with external criteria; P_CBCL_9 = Internalizing Problems; P_CBLC_10 = Externalizing Problems; P_CBCL_15 = Attention Deficit/Hyperactivity Problems; P_CBLC_16 = Oppositional Defiant Problems; P_CBCL_17 = Conduct Problems; Pc_agg = the first part of the Child-to-Parent Violence Questionnaire parent report; Cc_agg = the first part of the Child-to Parent Violence Questionnaire child report; aggF1 = Psychological Aggression factor; aggF2 = Physical Aggression factor; aggF3 = Financial Aggression factor; aggF4 = Control/Domain factor; Pc_rea = the second part of the Child-to-Parent Violence Questionnaire parent report; Cc_rea = the second part of the Child-to-Parent Violence Questionnaire child report; reaF1 = Instrumental Reason factor; reaF2 = Reactive Reason factor. (**a**) with CPV-Q-I parent report, (**b**) with CPV-Q-I child report, (**c**) with CPV-Q-II.

Concurrent validity of the C-CPV-Q-I and C-CPV-Q-II was supported by the positive correlations with the five external criterion measures. C-CPV-Q-I parent report had correlations with internalizing problems from small to moderate magnitudes (r = 0.18 to 0.39; *p* < 0.001); with externalizing problems from moderate to large magnitudes (r = 0.30 to 0.56; *p* < 0.001); with attention deficit/hyperactivity problems from small to moderate magnitudes (r = 0.20 to 0.45; *p* < 0.001); with oppositional defiant problems from moderate to large magnitudes (r = 0.31 to 0.54; *p* < 0.001); and with conduct problems from moderate to large magnitudes (r = 0.30 to 0.57; *p* < 0.001) (Figure 2a).

The C-CPV-Q-I child report, except for its physical aggression factor (r = −0.02 to 0.09; *p* = 0.813 to 0.143), had correlations with all external criterion measures from small to moderate magnitudes (r = 0.16 to 0.26; *p* = 0.046 to 0.001 [internalizing problems], r = 0.16 to 0.25; *p* = 0.003 to <0.001 [externalizing problems], r = 0.17 to 0.31; *p* = 0.008 to <0.001 [attention deficit/hyperactivity problems], r = 0.19 to 0.29; *p* = 0.014 to <0.001 [oppositional defiant problems], and r = 0.13 to 0.22; *p* < 0.001 [conduct problems]) (Figure 2b).

The C-CPV-Q-II parent report had correlations with all external criterion measures from moderate to large magnitudes (r = 0.39 to 0.65; *p* < 0.001), and the C-CPV-Q-II child report had correlations with all external criterion measures from small to moderate magnitudes (r = 0.17 to 0.28; *p* = 0.006 to <0.001) (Figure 2c).

## 4. Discussion

The results of the CFA in this study found that both child and parent versions of the C-CPV-Q-I contained four similar domains of CPV in adolescents with ADHD. The results of the present study supported the factor structure of the original CPV-Q, which was examined in 1386 adolescents and 1012 parents of adolescents [13,14]. Psychological aggression refers to behaviors intended to emotionally hurt parents (e.g., intimidating, belittling, and insulting) [41]. Physical aggression refers to acts such as throwing things at parents, spitting, kicking, punching [41]. Financial demand refers to behaviors such as stealing parents’ money and forcing parents to pay debts [41]. Control/domain over parents refers to the attitudes and behaviors intended to force parents to do what adolescents want [42]. Compared to other types of CPV, control/domain over parents is less likely to be noticed or evaluated. However, control/domain over parents can cause the loss of parental discipline and parents’ feelings of helplessness. The CPV-Q can be used to assess adolescents’ and parents’ perspectives on the four domains of CPV in adolescents with ADHD.

The results of the CFA in this study found that both child and parent versions of the C-CPV-Q-II contained two similar domains of the reasons for CPV, including instrumental and reactive reasons. CPV for instrumental reasons comes from adolescents’ intentions to achieve certain goals, while CPV for reactive reasons is adolescent’s reaction to certain parental discipline [21,22]. The present study expanded the original C-CPV-Q-II to 13 items to contain more clinically observed reasons of CPV, and the results of the CFA supported the two-factor structure of the expanded version. The CPV-Q can be used for clinical assessment of the reasons for CPV and help develop individualized intervention strategies for CPV in adolescents with ADHD. However, the metric invariance of the child and parent versions of the CPV-Q-II was not met, indicating that participants of different ages may interpret the CPV-Q-II differently. Because the original scale did not examine measurement invariance across various age groups when assessing the reasons for CPV among children and parents in the community, it is not possible to know if the results of this study occurred specifically in adolescents with ADHD and their parents. The results of this study suggested that the effect of participants’ age should be emphasized when using the CPV-Q to assess the reasons for CPV.

The result of the present study supported the cross-validity and concurrent validity of both C-CPV-Q-I and C-CPV-Q-II. Studies have found that the severity of ADHD symptoms and comorbid ODD significantly correlated with CPV in adolescents with ADHD [24,43,44]. Studies on adolescents in the community have found that adolescents who attack their parents have a higher proportion of comorbid ODD, ADHD, and behavioral deviance than other adolescents [45,46]. Studies have also found a cross-sectional association between adolescent depression and CPV [46], as well as the prediction of depression for CPV 6 months later [20]. The results of the present study indicated that CPV warrants routine surveys in adolescents with ADHD, especially among those with comorbid internalizing or externalizing problems.

The present study found that the entire C-CPV-Q-I child and parent versions had acceptable internal consistency, whereas the financial aggression factor had unsatisfactory internal consistency. The result was congruent with that of the original study [21]. This study also found that Items 6 and 12 in financial aggression on the child report had weak factor loadings. The financial aggression factor of the C-CPV-Q contains three items: “I have demanded my parents to buy me things even knowing they cannot afford it (Item 6)”; “I have acquired debts that my parents have had to pay (Item 7);” and “I have stolen money from my parents (Item 12)”. The unsatisfactory internal consistency of the financial aggression factor and weak factor loadings of Items 6 and 12 may indicate the heterogeneity of CPV contained in this factor. Further, Item 11 (“I have kicked, slapped, and/or punched my parents”) in physical aggression had a low factor loading. Other items in the physical aggression factor (Items 8 and 10) describe attacking parents with objects. This result shows that there is a difference in meaning between direct attacks and attacks with objects. Health professionals should be aware of this feature when using the CPV-Q.

### Limitations

This study has several limitations. First, adolescents with ADHD were recruited from outpatient clinics, where they were actively receiving pharmacological or psychological therapy. Future studies should examine the psychometric properties of the C-CPV-Q in adolescents with ADHD who are not receiving medical treatment. Additionally, the exclusion of adolescents with comorbid conditions such as autism spectrum disorder, bipolar disorder, or schizophrenia, while methodologically justifiable, may limit the ecological validity of the findings. Given the high rate of comorbidity in ADHD, excluding these groups may reduce the applicability of the results to real-world ADHD populations. Second, the temporal associations between CPV and internalizing or externalizing problems could not be determined because of the cross-sectional design of this study. Third, this study did not examine whether the father and mother of adolescents with ADHD have different reports of CPV. Fourth, measurement invariance was not tested for the CPV-Q because of the small sample sizes for fathers (*n* = 65) and girls (*n* = 45). Future studies are thus needed to increase the father/girl samples to evaluate the measurement invariance of CPV-Q across gender subgroups.

## 5. Conclusions

This study translated the CPV-Q into the Chinese version for assessing child and parent reports of CPV in adolescents with ADHD. This study found that the Chinese version of the child and parent reported CPV-Q had a four-factor structure in assessing CPV types and a two-factor structure in assessing the reasons for CPV, acceptable internal consistency, and moderate positive correlations with internalizing, externalizing, attention deficit/hyperactivity, oppositional defiant, and conduct problems. The results indicate that the child and parent versions of the C-CPV-Q are valid instruments for assessing a wide variety of CPV behaviors and the reasons for the violence among adolescents with ADHD from the adolescents’ and parents’ perspectives. Knowing both perceptions of CPV will facilitate the development of intervention programs for CPV [22]. CPV warrants routine surveys in adolescents with ADHD, especially among those with comorbid internalizing or externalizing problems.

## Figures and Tables

**Table 1:** Participants’ characteristics (N = 247).

	Mean (*SD*) or *n* (%)
Parent gender	
Male	65 (26.3)
Female	182 (73.7)
Child gender	
Boy	206 (83.4)
Girl	41 (16.6)
Parent age (year)	46.40 (6.41)
Child age (year)	13.21 (2.03)
Parent educational level	
High school or below	80 (32.4)
College or above	167 (67.6)

**Table 2:** Factor loadings and fit indices in the confirmatory factor analysis of the first part of the Child-to-Parent Violence Questionnaire (CPV-Q-I).

Factor	Factor Loading or (ω/α)	
Item	Parent Report	Child Report
**Psychological aggression**	(0.88/0.85)	(0.81/0.75)
Item 1	0.585	0.656
Item 2	0.908	0.656
Item 3	0.913	0.738
Item 4	0.568	0.536
**Physical aggression**	(0.82/0.76)	(0.73/0.62)
Item 8	0.765	0.636
Item 10	0.485	0.654
Item 11	0.733	0.302
**Financial aggression**	(0.45/0.41)	(0.46/0.37)
Item 6	0.322	0.304
Item 7	0.664	0.534
Item 12	0.368	0.313
**Control/domain**	(0.81/0.79)	(0.74/0.64)
Item 5	0.804	0.512
Item 9	0.567	0.417
Item 13	0.765	0.486
Item 14	0.622	0.614
**Fit indices for the entire model**		
χ^2^ (df)/*p*-value	79.834 (69)/0.175	67.867 (76)/0.736
Comparative fit index	0.938	1.000
Tucker–Lewis index	0.918	1.191
RMSEA (90% CI)	0.025 (0.000, 0.047)	0.000 (0.000, 0.028)
SRMR	0.075	0.080
ω/α	0.89/0.85	0.81/0.76

Note: RMSEA = root mean square error of approximation; CI = confidence interval; SRMR = standardized root mean square residual.

**Table 3:** Factor loadings and fit indices in the confirmatory factor analysis of the second part of the Child-to-Parent Violence Questionnaire (CPV-Q-II).

Factor	Factor Loading or (ω/α)	
Item	Parent Report	Child Report
**Instrumental reasons**	(0.91/0.82)	(0.82/0.78)
Item 1	0.446	0.501
Item 2_1	0.546	0.384
Item 2_2	0.588	0.654
Item 3	0.808	0.631
Item 4	0.800	0.680
Item 5	0.647	0.698
**Reactive reasons**	(0.81/0.75)	(0.84/0.78)
Item 6	0.537	0.833
Item 7	0.414	0.398
Item 8	0.581	0.458
Item 9	0.786	0.764
Item 10	0.592	0.506
Item 11	0.374	0.473
Item 12	0.483	0.354
**Fit indices for the entire model**		
χ^2^ (df)/*p*-value	84.572 (59)/0.016	67.817 (59)/0.202
Comparative fit index	0.928	0.938
Tucker–Lewis index	0.905	0.918
RMSEA (90% CI)	0.042 (0.019, 0.061)	0.025 (0.000, 0.048)
SRMR	0.078	0.070
ω/α	0.89/0.86	0.87/0.84

Note: RMSEA = root mean square error of approximation; CI = confidence interval; SRMR = standardized root mean square residual.

**Table 4:** Measurement invariance findings across age subsamples for the four measures.

	Measure			
	CPV-Q-I Child Report	CPV-Q-I Parent Report	CPV-Q-II Child Report	CPV-Q-II Parent Report
Configural vs. Metric				
CFI difference	0.000	0.000	0.147	0.041
RMSEA difference	0.000	0.000	0.066	0.037
Metric vs. Scalar				
CFI difference	0.000	0.000	0.006	0.002
RMSEA difference	0.000	0.000	0.004	0.008

Note: CPV-Q-I = the first part of the Child-to-Parent Violence Questionnaire; CPV-Q-II = the second part of the Child-to-Parent Violence Questionnaire; CFI = comparative fit index; RMSEA = root mean square error of approximation.

## Data Availability

The data are available upon reasonable request to the corresponding authors.
